# P68 Identifying the Content and Context of Pain Within Education and Training of General Practitioners

**DOI:** 10.1093/rap/rkac067.068

**Published:** 2022-09-28

**Authors:** Yan Jun Ong, Maisun Elftise, Janet McDonagh, Rebecca Lee

**Affiliations:** University of Manchester Medical School, Manchester, United Kingdom; Portfolio GP, Coventry and Warwickshire Training Hub, Coventry, United Kingdom; Centre for Epidemiology Versus Arthritis University of Manchester, Manchester, United Kingdom; NIHR Biomedical Researh Centre, Manchester Unviersity Hospitals NHS Trust, Manchester, United Kingdom; Centre for Epidemiology Versus Arthritis University of Manchester, Manchester, United Kingdom; NIHR Biomedical REsearch Centre Manchester University Hospitals, Manchester, United Kingdom

## Abstract

**Introduction/Background:**

Chronic musculoskeletal pain is prevalent in over 33% of children and adolescents. In order to manage this pain appropriately, healthcare professionals need to have the knowledge and skills to do so. Sub-optimal coverage has been reported in undergraduate curricula and even in specialist postgraduate paediatric rheumatology curricula. Pain is a frequent presentation in primary care but the coverage of pain in the core curricula of general practice training is not known. The aim of this study was to identify pain-related content within the curricula and understand the context in which these terms present in.

**Description/Method:**

A directed search within the Royal College of General Practitioners (RCGP) was executed in April 2022. A systematic hand search was further carried out in early April to identify resources for this article. This was done using leading web search engines in the UK: Google, Microsoft Bing and Yahoo. The keywords for these searches included: ‘GP’, ‘general practitioners’, ‘curriculum’, curricula’, ‘training’ and ‘guidelines’. A number of general practitioners across the UK (N = 3) were also approached to further help with identifying possible documents guiding the training and curricula of general practitioners for analysis. After analyses were done, an informal interview was carried out with a general practitioner in order to ensure the validity of the results and findings.

A quantitative summative content analysis was performed on the materials identified. This was done by calculating the frequencies of pre-determined key terms: ‘pain’, ‘pain management’, ‘acute pain’, ‘chronic pain’ and ‘pain relief’. This search was done in such a way that it was able to account for terms that were used separately, such as ‘pain’ and ‘management’, within a sentence to be coded as ‘pain-management’. Additional non pre-determined key terms, such as ‘pains’, ‘painful’, ‘long term pain’, ‘pain perception’ and ‘alleviate pain’, were also identified. After these key terms were identified, the weighted frequencies of terms were calculated. A qualitative summative content analysis was then carried out to arrange key phrases into themes which could describe the context in which key terms were presented. This study used a latent approach where each phrase with pain terms was considered in relation to its salient underlying meaning. NVivo software (QRS International, Warrington, UK) was used to facilitate analysis.

**Discussion/Results:**

9 resources were identified but 7 were excluded as they were not part of the core curricula of the specialty. The 2 documents used for analyses were: The RCGP Curriculum *Being a General Practitioner* (2020) and The RCGP Curriculum *The Curriculum Topic Guides* (2020). Key terms were only present in the latter document with 113 key terms, accounting for 0.1% of the document in total. Within these 113 pain terms, 7 were part of case discussions, comprising merely 0.06% of the pain terms. 5 of the 113 pain terms (0.04%) were within the phrase ‘chronic pain’. There were no phrases of ‘acute pain’ found within the document. Further qualitative analysis was carried out and identified 7 main themes. (i) Definition of pain: keywords were found to predominantly be in the context of acute presentations. Pain in the context of chronic pain was more often in examples of long-term conditions and/or as part of multimorbidity. (ii) Body systems associated with and/or affected by pain e.g. musculoskeletal, cardiovascular, abdominal, neurological, respiratory, genitourinary and reproductive systems. (iii) The biopsychosocial model of pain: phrases focussed on the appropriate care in order to maximise patients’ psychosocial wellbeing in the context of underlying diagnoses. (iv) Recognition and communication of pain: identifying the cause of pain and how pain is expressed and perceived by both patient and general practitioners. (v) Impact of pain on patients, carers and families. (vi) Pain in age groups: pain within children was mentioned the most, followed by adults, then adolescents more specifically. (vii) Pain management: pain in the context of relief, management, care and alleviation. It was also noted that reference to pain was made in the context of case discussions (reflective learning practice) and formative assessments (Applied Knowledge Test, Clinical Skills and Workplace Based Assessments).

**Key learning points/Conclusion:**

This research has identified gaps in the content and context of pain within the curricula for general practice postgraduate training in the UK. Currently, there is limited reference to pain in the general practice curricula generally. When compared to the core pain curricula proposed by the International Association for the Study of Pain (IASP), there was limited coverage of general practitioners' understanding of the importance of fundamental concepts of pain, environmental influences, pain assessment and measurement, and pain management, as well as unique pain assessment and management approaches for people with additional needs. IASP describe these core components of pain education as pertinent to all healthcare professionals potentially seeing patients with chronic pain in their clinical practice. In view of the prevalence of chronic pain in the general population (in adults and in children/young people), these discrepancies should be reviewed and addressed, thereby improving the integration of pain education into postgraduate training programmes in the future. This is especially important given that undergraduate training in pain is equally as limited, as identified in others' past research. By improving the knowledge, skills and confidence of general practitioners in pain assessment, communication and management approaches, this will ultimately impact upon the long-term outcomes of individuals living with chronic pain across the life course.

